# Niacinamide mitigated the acute lung injury induced by phorbol myristate acetate in isolated rat's lungs

**DOI:** 10.1186/1423-0127-19-27

**Published:** 2012-03-01

**Authors:** Chia-Chih Lin, Nan-Kuang Hsieh, Huey Ling Liou, Hsing I Chen

**Affiliations:** 1 Department of Physical Education and Kinesiology, National Dong Hwa University, Hualien, Taiwan; 2 Department of Family Medicine, Tao-Yuan General Hospital, Department of Health, Executive Yuan, Taoyuan, Taiwan; 3 Department of Pathology, China Medical University, Taichung, Taiwan; 4 Department of Nursing, Taipei Veterans General Hospital, Taipei, Taiwan; 5 Institute of Physiological and Anatomical Medicine, Tzu Chi University, Hualien, Taiwan

**Keywords:** phorbol myristate, niacinamide, acute lung injury, isolated perfused lungs, neutrophil-derived mediators, nitric oxide

## Abstract

**Background:**

Phorbol myristate acetate (PMA) is a strong neutrophil activator and has been used to induce acute lung injury (ALI). Niacinamide (NAC) is a compound of B complex. It exerts protective effects on the ALI caused by various challenges. The purpose was to evaluate the protective effects of niacinamide (NAC) on the PMA-induced ALI and associated changes.

**Methods:**

The rat's lungs were isolated *in situ *and perfused with constant flow. A total of 60 isolated lungs were randomized into 6 groups to received Vehicle (DMSO 100 μg/g), PMA 4 μg/g (lung weight), cotreated with NAC 0, 100, 200 and 400 mg/g (lung weight). There were 10 isolated lungs in each group. We measured the lung weight and parameters related to ALI. The pulmonary arterial pressure and capillary filtration coefficient (K_fc_) were determined in isolated lungs. ATP (adenotriphosphate) and PARP [poly(adenosine diphophate-ribose) polymerase] contents in lung tissues were detected. Real-time PCR was employed to display the expression of inducible and endothelial NO synthases (iNOS and eNOS). The neutrophil-derived mediators in lung perfusate were determined.

**Results:**

PMA caused increases in lung weight parameters. This agent produced pulmonary hypertension and increased microvascular permeability. It resulted in decrease in ATP and increase in PARP. The expression of iNOS and eNOS was upregulated following PMA. PMA increased the neutrophil-derived mediators. Pathological examination revealed lung edema and hemorrhage with inflammatory cell infiltration. Immunohistochemical stain disclosed the presence of iNOS-positive cells in macrophages and endothelial cells. These pathophysiological and biochemical changes were diminished by NAC treatment. The NAC effects were dose-dependent.

**Conclusions:**

Our results suggest that neutrophil activation and release of neutrophil-derived mediators by PMA cause ALI and associated changes. NO production through the iNOS-producing cells plays a detrimental role in the PMA-induced lung injury. ATP is beneficial, while PARP plays a deteriorative effect on the PMA-induced ALI. NAC exerts protective effects on the inflammatory cascade leading to pulmonary injury. This B complex compound may be applied for clinical usage and therapeutic regimen.

## Background

Niacinamide or nicotinamide is a compound of the soluble B complex. It exerts inhibitory effects on the poly (ADP-ribose) synthase (PARS) or poly (ADP-ribose) polymerase (PARP). The nuclear enzyme can be activated by strand breaks in DNA caused by reactive oxygen species and peroxynitrite [1-3]. PARP is cytotoxic by massive depletion of intracellular nicotinamide adenine dinucleotide (NAD^+^) and adenosine triphosphate (ATP). Inhibition of PARP activity reduces the ischemia-reperfusion injury of the heart, skeletal muscle, and brain [3-5]. In addition, PARP inhibition with a specific blocker, 3-aminobenzamide attenuates the acute lung injury induced by endotoxin [6].

The inhibitory effects of niacinamide or its related substances, nicotinamide and nicotinic acid, on the PARP activity are protective to cell damage caused by oxidative stress [7-10]. Our laboratory has reported that PARS or PARP inhibition with niacinamide attenuates the ischemia-reperfusion hepatic injury [11].

Phorbol myristate acetate (12-*O*-tetradecanoyl-phorbol-13-acetate, PMA), an ester derivative from croton oil has been used to induce acute lung injury (ALI) in animal experiments [12,13]. PMA is a strong neutrophil activator. It leads to neutrophil activation and recruitment. Consequently, release of neutrophil mediators such as neutrophil elastase and other factors results in severe acute lung injury [14-17].

The present study was designed to test the protective effects of niacinamide on the PMA-induced ALI and associated changes. The beneficial effects of niacinamide were assessed in a dose-dependent fashion using various doses. We also aimed to elucidate the mechanism of action of this B-complex agent.

## Methods

### Animal preparation

We used male Spaque-Dawley (SD) rats, 12-14 wk-old, weighing 360-380 g. The animals were obtained from the National Animal Center and housed in the University Laboratory Animal Center with good environment control. The animal experiment was approved by the University Committee of Laboratory Animal Care and Use, and followed the guidelines of the National Animal Research Center. The room temperature was maintained at 21 ± 1°C under a 12/12 hr light/dark regimen. Food and water were provided *ad libitum*.

### Isolation and perfusion of the lung *in Situ*

We followed the procedures for the preparation of isolated and perfused rat's lungs *in situ *[18,19]. Rats were anesthetized with an intraperitioneal injection of pentobarbital (40 mg/kg) and intubated with an endotracheal tube. A rodent ventilator provided ventilation with a mixture of 95% room air and 5% carbon dioxide. The respiratory rate and tidal volume were 60-65 breaths/min and 2-3 ml, respectively. The inspiratory and expiratory pressures were 5 and 1 cm H_2_O. A vertical incision was made along the midline of the thorax. Heparin (1 U/g) was then injected into the right ventricle. An afferent line (silicon tubing) was then inserted into the pulmonary arterial trunks via the right ventricle, while an efferent line (silicon tubing) was inserted into the left atrium via the left ventricle. Blood (10-15 ml) was collected for later use.

The pulmonary trunk and the aorta were then tied off. The isolated perfused lungs were left *in situ*, and the whole rat was placed on an electronic balance. The digital signals of the electronic balance were converted to analog signals by a digital-to-analog converter and were recorded on a polygraph recorder. Weight changes were precalibrated on the electronic balance before preparation for the experiment. In this isolated lung preparation, we have verified that the changes in body weight (BW) reflect the lung weight (LW) changes [18,19].

The isolated lungs were perfused with heparinized blood (10 ml) with Krebs-Heseleit balanced salt solution (5 ml). The perfusion system included a venous reservoir and a roller pump. The perfusate was circulated via the roller pump to maintain a constant flow. The venous blood was diverted via the efferent line into the reservoir. The later was placed in a 38°C water bath for constant temperature.

Pulmonary arterial pressure (PAP) and venous pressure (PVP) were measured from sideports connected to the afferent and efferent tubings. The lungs were perfused with a constant flow (10-14 ml/min). Flow rate was adjusted to maintain the initial PAP at 15-16 mmHg. The PVP was kept at 0-1 mmHg by adjusting the height of efferent outflow tubing. The changes in PAP at a constant-flow condition reflect the changes in pulmonary vascular resistance.

### Lung weight (LW), LW/body weight ratio (LW/BW) and LW gain (LWG)

Lung weight (LW) was obtained from 20 euthanized rats by an intravenous sodium pentobarbital (100 mg/kg). The initial LW was estimated from an equation relating to the body weight (BW) [18,19]. The LW was then plotted against BW for a regression equation:

$$\text{LW}\left(\text{g}\right)\;=\;0.00\text{15 }\times \text{ BW}\left(\text{g}\right)\;+\;0.0\text{34}$$

LWG was obtained by the increase in LW and also calculated as:

LWG=final LW-initial LW

### Microvascular permeability (K_fc_)

Capillary filtration coefficient (K_fc_) as an index of microvascular permeability was calculated from the increase in LW produced by an elevation in PVP. The K_fc _was defined as the initial weight gain rate (g/min) divided by PVP (10 cm H_2_O) and LW, expressed as g/min/cmH_2_O/100 g. During the experiment, PVP was rapidly elevated by 10 cm H_2_O for 7 mins to measure K_fc_. This hydrostatic challenge elicited a biphasic increase in LW: an initial rapid component, followed by slow and steady component. The slow component of the weight gain was plotted on a semilog scale as a function of time. The capillary filtration rate was obtained by extrapolating the slow component of the weight gain back to zero time [18,19].

### Exhaled NO

An increase in exhaled NO concentration has been used as an early marker of lung inflammation or injury [20,21]. We measured the NO concentration in expired air. A specimen of exhaled air (300 ml in 30 min) was suctioned into a gas purge chamber preciously evacuated to remove oxygen. The NO concentration was rapidly determined after air collection. The measurement of NO with a chemiluminescence analyzer (Sievers 270B NOA; Sievers Institute, Denver, CO, USA) was based on the principle that NO interacts with ozone to generate chemiluminescent light. The chemiluminescence is directly proportional to the NO level. In addition to a photomultiplier tube, an ozone generator and a gas chamber were included. The ozone generator was used to produce ozone internally. The exhaled NO was measured every 30 min after introduction of PMA into the lung perfusate. It reached its peak depending on the experimental conditions. The peak value was taken as the NO concentration.

### Protein concentration in bronchoalveolar lavage (PCBAL) and Evans blue leakage

After the experiment, lungs were lavaged twice with saline (2.5 ml per lavage). Lavage samples were centrifuged at 1,500 g at room temperature for 10 min. The PCBAL was determined with a spectrophotometer by measuring the change in absorbance at 630 nm after the addition of bromocresol green [18,22]. Five mins before the end of the experiment, Evans blue dye (1 mg) was given into the lung perfusate. The Evans blue content in lung tissue was determined spectrophotometrically at an optical density 620 nm [22].

### Nitrate/nitrite, methyl guanidine, tumor necrosis factor_α _and interleuin-1_β_

Samples (0.5 ml) were taken from the lung perfusate 1 hr before and at various time points after PMA administration. The samples were centrifuged at 3,000 g for 10 mins. The supernatant was used for determination of nitrate/nitrite with high-performance chromatography [23,24]. The formation of methyl guanidine (MG) has been identified as an index of hydroxyl radical production [25]. It was determined with its fluorescence spectrum (Jasco 821-FP, Spectroscopic Co., Tokyo, Japan). The emission maximum was set at 500 nm and the excitation maximum at 398 nm. The assay was calibrated with authentic MG (Sigma M0377). Tumor necrosis factor_α _(TNF_α_) and interleukin-1_β _(IL-1_β_) were measured with antibody enzyme-linked immunosorbent assays (ELISAs) with a commercial antibody pair, recombinant standards, and a biotin-streptavdin-peroxidase detection system (Endogen, Rockford, IL, USA). All agents, samples, and working standards were prepared at room temperature according to the manufacturer's directions. The optical density was measured at 450/540 nm wavelengths by automated ELISA readers.

### Neutrophil elastase, myeloperoxidase, and malondialdehyde activity

The neutrophil elastase (NE) was determined with a synthetic substrate, N-methoxysuccinyl-Ala-Ala-Pro-Val-p-nitroanilide as described previously [15,18]. In brief, samples were incubated in 0.1 M Tris-HCl buffer (pH 8.0) containing 0.5 M Nacl and 1 mM substrate at 37°C for 24 hrs. After incubation, p-nitroanilide release was measured spectrophotometrically at 450 nM and considered NE activity.

To measure the myeloperoxidase (MPO) activity in lung perfusate, the samples were mixed with 2 ml of potassium phosphate buffer (50 mM, pH 6.0) containing 0.5% cetyltrimethylammonium bromide and were centrifuged at 2,500 g for 10 mins at 4°C. The supernatant was diluted with dilution buffer, then mixed with an assay buffer composed of 0.00107% H_2_O_2 _in potassium phosphate buffer and o-dianisidine. The reaction mixture was incubated at room temperature. The change in absorbance at 450 nm over 1 min was detected spectrophotometrically. The MPO activity was expressed as units per ml of lung perfusate using the absorbance of MPO standard (Elastine Products, Detroit, MC., USA). The procedures were basically followed those by Kinoshita et al. [26].

Malondialdehyde (MDA) was measured by thiobarbituric and reaction. The principle of the method depends on the development of pink color produced by the interaction of barbituric acid with MDA as a result of lipid peroxidation. Tetraetoxypropane was used as standard [27,28].

### ATP content

Lungs were harvested after the experiments. A BioOrbit ATP Assay kit (Bio-Orbit Oy, Turku, Finland) was used to determine the lung ATP content with bioluminescence technique. The assay was based on quantitative measurement of a stable level of light as a result of an enzyme reaction catalysed by luciferase. Under the effect of luciferase, the luminescence evoked by ATP and luciferin interaction was recorded photometrically after amplification by a photomultiplier. The sensitivity of ATP was in a nanomolar range. The luciferin-luciferase reagent was used according to the manufacturer's manual. ATP served as the standard. The test procedures have been described previously [11,29].

### PARP activity

PARP activity in the harvested lung tissue was measured with a commercially available assay kit (Genzyme Diagnostics, Cambridge, MA, USA). Lung tissue samples were placed on ice in 2 mL buffer containing 50 mM Tris Cl (pH 8.0), 25 mM MgCl_2 _and 0.1 mM phenylmethylsulfonyl fluoride. The samples were homogenized for 30 s and then sonicated for 20 s using an ultrasonic homogenizer. The suspension was centrifuged at 3,000 × g for 5 min at 4°C. Supernatant containing 20 μg protein, PARS buffer, 1 mM NAD, 2 μCi ^32^P-labelled NAD (1 μCi/μL) and distilled water was mixed in a microcentrifuge tube. The reaction was allowed to continue at room temperature for 1 min and was stopped by adding 900 μL of tricarboxylic acid. Enzyme activity was determined by measuring the incorporation of radiolabelled NAD as PARP catalysed the poly (ADP) ribosylation of proteins. The labeled ADP was determined by scintillation counting after tricarboxylic acid precipitation onto a filter. The procedures and calculation of PRRP activity were carried out according to those described by Pulido et al. [30].

### Detection of iNOS and eNOS mRNA in lung tissue

Real-time polymerase chain reaction (RT-PCR) was employed for the detection of iNOS and eNOS mRNA expression. PCR primers and TaqMan-MGB probes were designed using Primer Express V.2.0 software (Applied Biosystems Inc., Foster, CA, USA) based on the sequences from GenBank. TaqMan-MGB probes were labeled with 6-carboxy-fluorescein (FAM) as the reporter dye. Real-time PCR was performed in a two-step process: In the first step, sample RNA (100 ng) was reverse-transcribed with 50 ng random hexamers in a volume of 20 μl using 200 U of Superscript III reverse transcriptase and 40 U of RNaseOUT recombinant RNase inhibitor (both from Invitrogen, Carlsbad, CA, USA). In the second, real-time PCR was carried out in a MicroAmp Optical 96-well plate using TaqMan Master Mix (Applied Biosystems Inc.), with 5 μl cDNA in each well. PCR reactions were monitored in real time using the ABI PRISM 7000 Sequence Detector (Applied Biosystems Inc. Foster, CA, USA). The thermal cycling conditions for real-time PCR were a) 50°C for 2 mins, b) 95°C for 10 mins, c) 40 cycles of melting (95°C, 15 sec) and d) annealing/extension (60°C, 60 sec).

### Immunofluorescent stain

Immunofluorescent staining was used to detect the activities of nitrotyrosine and iNOS in lung tissue using specific polyclonal antibodies as described previously [31,32].

### Lung pathology and immunohistochemical examinations

Lung tissue was fixed in 10% formaldehyde for 24 hrs and then rinsed with tap water to remove formaldehyde. For light microscopic examination, lung tissue was dehydrated with graded alcohol and then embedded in paraffin at 60°C. A series of microsections (5 μm) was stained with hematoxylin and eoxin. For quantification of lung injury score, we employed a modified grading method reported previously [21,28]. Various degree of lung injury score (LIS) were assessed as follows: degree 0, 1, 2 and 3 for no, mild, moderate and severe edema, respectively. For inflammatory cell infiltration, the scoring was similar to the edema extent: 0-3 for no, mild, moderate and severe cell infiltration. The histopathological assessment was performed in a blind fashion by several laboratory assistants. The individual scores were added together to obtain a final score, ranging from 0-6.

Antigen retrieval immunohistochemical stain was used to identify the source of inducible NO synthase (iNOS) in lung cells [33,34].

### Administration of PMA and niacinamide (NAC)

PMA (4 μg/g) and NAC at various doses were added into the lung perfusate via the venous reservoir [35,36]. NAC was given simultaneously with PMA [20,37,38]. Examinations of biochemical factors were taken before administration of PMA and NAC, and at the end of the experiments.

### Experimental protocol

A total of 60 isolated lungs were randomized into 6 groups to receive Vehicle (DMSO 100 μg/g), and PMA 4 μg/g (lung weight) cotreatment with NAC 0, 100, 200 and 400 mg/g (lung weight). There were 10 isolated lungs in each group. The doses of PMA and NAC were used in accordance with previous studies [12,13,20]. The experiments were observed for 120 mins.

### Statistical analysis

All data were expressed as mean ± SEM. Comparisons within and among groups were made using one-way analysis of variance with repeated measures, followed a *post hoc *comparison with Newman-Keuls test. Differences were considered to be statistically significant at *p *< 0.05.

## Results

### Lung weight/body weight (LW/BW) ratio, LW gain (LWG), exhaled NO and protein concentration in bronchoalveolar lavage (PCBAL)

**Figure 1:**
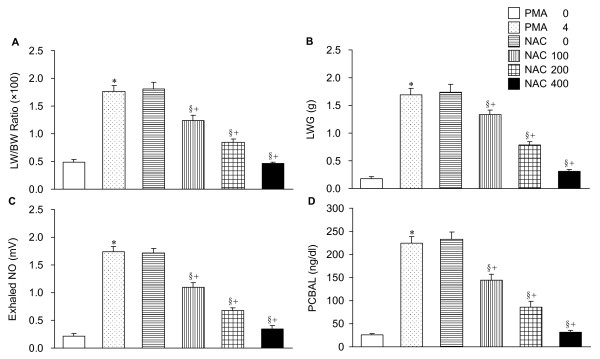
**The lung weight (LW) to body weight (BW) ratio (A), LW gain (LWG, B), exhaled nitric oxide (NO, C), and protein concentration in bronchoalveolar lavage (PCBAL, D)**. Phorbol myristate acetate (PMA) significantly increased these lung variables. Administration of niacinamide (NAC) attenuated the PMA-induced changes in a dose-dependent manner. PMA at a dose of 4 μg/g (PMA 4) (lung weight) was given into the lung perfusate. PMA 0 denotes the control lungs without receiving PMA. NAC 0, 100, 200, and 400 indicate the doses of NAC at mg/g (lung weight). *p < 0.05 PMA 4 vs. PMA 0. ^§^*p *< 0.05 vs. NAC 0 (dose effects of NAC). ^+^*p *< 0.05 vs. the values of PMA 4. There were 10 isolated lung preparations in each group.

Figure 1 illustrates the PMA effects on the LW/BW ratio, LWG, exhaled NO and PCBAL. Cotreatment with NAC mitigated the PMA-induced changes. The protective effects of NAC were dose-dependent.

### Pulmonary arterial pressure (PAP) and capillary filtration coefficient (K_fc_)

**Figure 2:**
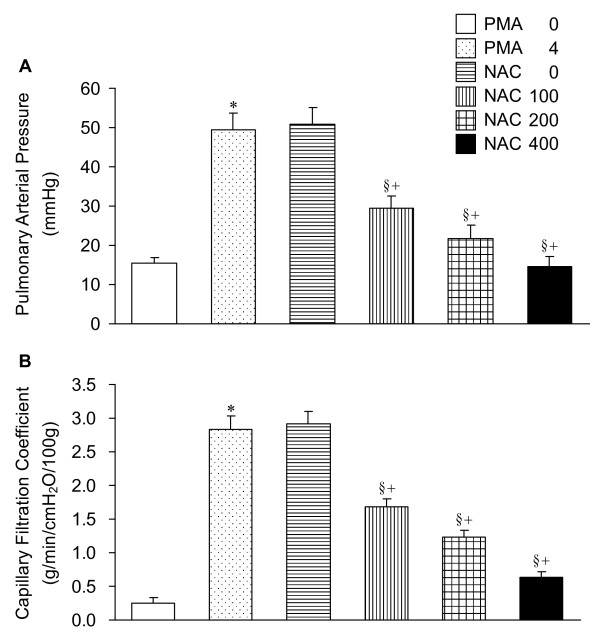
**Pulmonary arterial pressure (PAP) and capillary filtration coefficient (K_fc_) in isolated lungs**. The denotations and statistical symbols are the same as Figure 1.

In isolated lungs, PMA caused increases in PAP and K_fc_. The PMA-induced pulmonary hypertension and increased microvascular permeability were attenuated by NAC. The protection was dependent on the NAC doses (Figure 2).

### Nitrate/nitrite, methyl guanidine, tumor necrosis factor_α _and interleukin-1_β_

**Figure 3:**
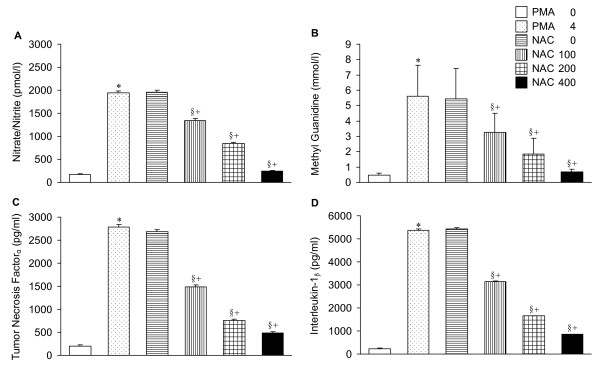
**Concentration of nitrate/nitrite (A) methyl guanidine (B), tumor necrosis factor_α _(C), and interleukin-1_β _(D) in lung perfusate**. Note the dose-dependent elevation in these factors by PMA, and the depression by coadministration with NAC. Abbreviations and statistical symbols are the same as Figure 1.

PMA caused increases in these biochemical factors. Cotreatment with NAC reversed the PMA-induced changes. The effects of NAC were dose-dependent (Figure 3).

### ATP and PARP activities in lung tissues

**Figure 4:**
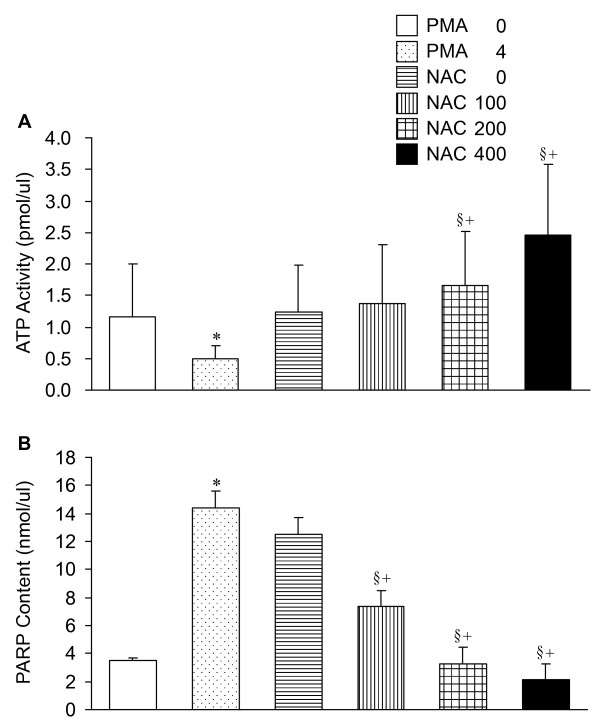
**ATP (A) and PARP (B) activities in lung tissue**. PMA resulted in a dose-dependent decrease in ATP content with increase in PARP. Cotreatment with NAC restored the ATP while reduced the PARP. Abbreviations and statistical symbols are the same as Figure 1.

Figure 4 displays the effects of PMA and NAC on the ATP and PARP contents in lung tissues. PMA caused decrease in ATP and increase in PARP in lung tissues. Cotreatment with NAC mitigated the PMA-induced changes. The protection was dose-dependent.

### Expression of iNOS and eNOS mRNA

**Figure 5:**
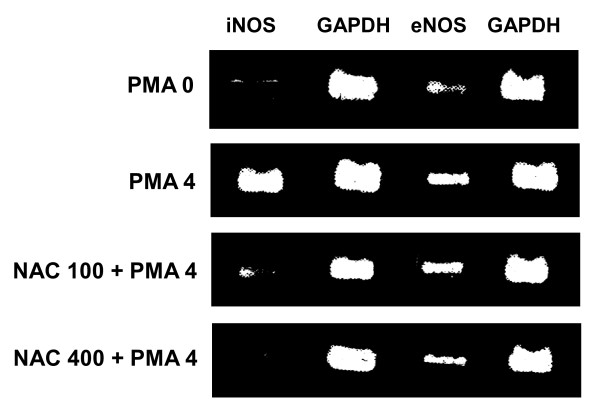
**The expression of inducible and endothelial nitric oxide synthase mRNA (iNOS and eNOS) using real-time RT-PCR**. GAPDH serves as internal contrast. PMA upregulated iNOS significantly and eNOS modestly. NAC treatment reduced the iNOS and eNOS expression. To avoid redundance, only the effects of NAC 100 and 400 mg/g on PMA 4 μg/g are shown. PMA 0 serves as control.

The expression of iNOS and eNOS in lung tissues was shown on Figure 5. PMA upregulated the iNOS and eNOS. The expression of iNOS was more obvious than eNOS. Coadministration with NAC attenuated the iNOS and eNOS expression. The effects were dose-dependent (Table 1).

**Table 1:** The expression of inducible and endothelial nitric oxide synthase (iNOS and eNOS) in lung tissue

		iNOS/GAPDH	eNOS/GAPDH
PMA	0	0.23 ± 0.02	0.44 ± 0.04
PMA	4	3.42 ± 0.12*	2.84 ± 0.14*
NAC	0	3.51 ± 0.14	3.02 ± 0.11
NAC	100	0.34 ± 0.04^§+^	2.01 ± 0.09^§+^
NAC	200	0.19 ± 0.06^§+^	1.46 ± 0.07^§+^
NAC	400	0.21 ± 0.04^§+^	0.62 ± 0.08^§+^

Values are means ± SEM (n = 10). The data include 10 isolated lung preparations. *p < 0.05 PMA 4 vs. PMA 0. ^§^*p *< 0.05 vs. NAC 0 (dose effects of NAC). ^+^*p *< 0.05 vs. the values of PMA 4. PMA, phorbol myristate acetate; and NAC, niacinamide. The symbols 0 and 4 denote the dose of PMA at μg/g (lung weight), and 0, 100, 200 and 400 of NAC at mg/g (lung weight). The expression of glyceraldehyde phosphate dehydrogenase (GAPDH) was used as an internal standard.

### NE, MPO and MDA

In lung perfusate, PMA caused increases in NE, MPO and MDA. NAC at different doses reduced the PMA-induced changes in these mediators. The effects of reduction depended on the doses (Table 2).

**Table 2:** Neutrophil elastase (NE), myeloperoxidase (MPO), and malondialdehyde (MDA)

		NE (nmol/ml)	MPO (unit/ml)	MDA (nmol/ml)
PMA	0	0.22 ± 0.03	4.23 ± 0.08	33.51 ± 2.56
PMA	4	21.42 ± .018*	49.44 ± 0.31*	486.51 ± 12.42*
NAC	0	22.14 ± 0.16	51.62 ± 0.33	512.46 ± 14.36
NAC	100	10.21 ± 0.14^§+^	24.36 ± 0.26^§+^	246.82 ± 4.92^§+^
NAC	200	4.22 ± 0.08^§+^	12.12 ± 0.12^§+^	112.34 ± 4.86^§+^
NAC	400	0.36 ± 0.04^§+^	5.63 ± 0.09^§+^	46.74 ± 0.24^§+^

Values are means ± SEM (n = 10). The data collections, abbreviations and statistical symbols are the same as Table 1.

### Nitrotyrosine and iNOS immunofluorescence

**Figure 6:**
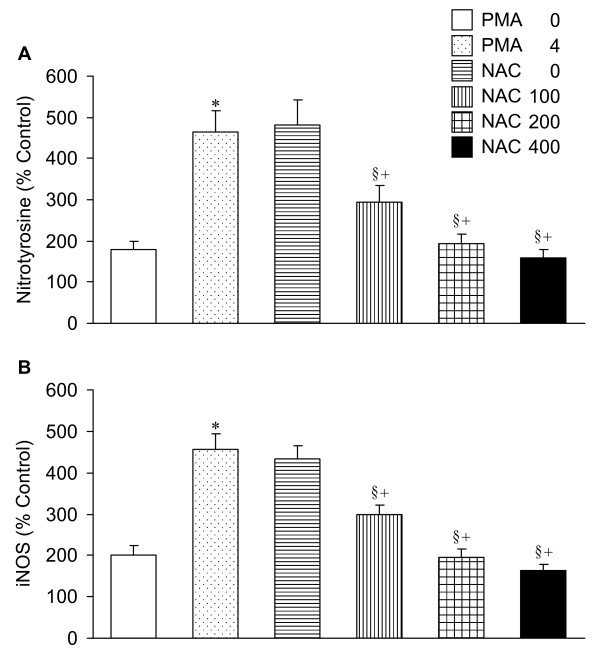
**Immunofluorescence of nitrotyrosine and iNOS in lung tissue**. Note the significant elevation of nitrotyrosine and iNOS after PMA. Coadministration of NAC reduces the PMA-induced increases in nitrotyrosine and iNOS in a dose-dependent manner. The abbreviations and statistical symbols are the same as Figure 1.

PMA elicited significant increases in nitrotyrosine and iNOS immunofluorescent activities. The enhancement of nitrotyrosine and iNOS in lung tissues following PMA was attenuated by co-treatment with NAC. The effects of NAC were dose dependent (Figure 6).

### Pulmonary pathology and immunohistochemical examinations

**Figure 7:**
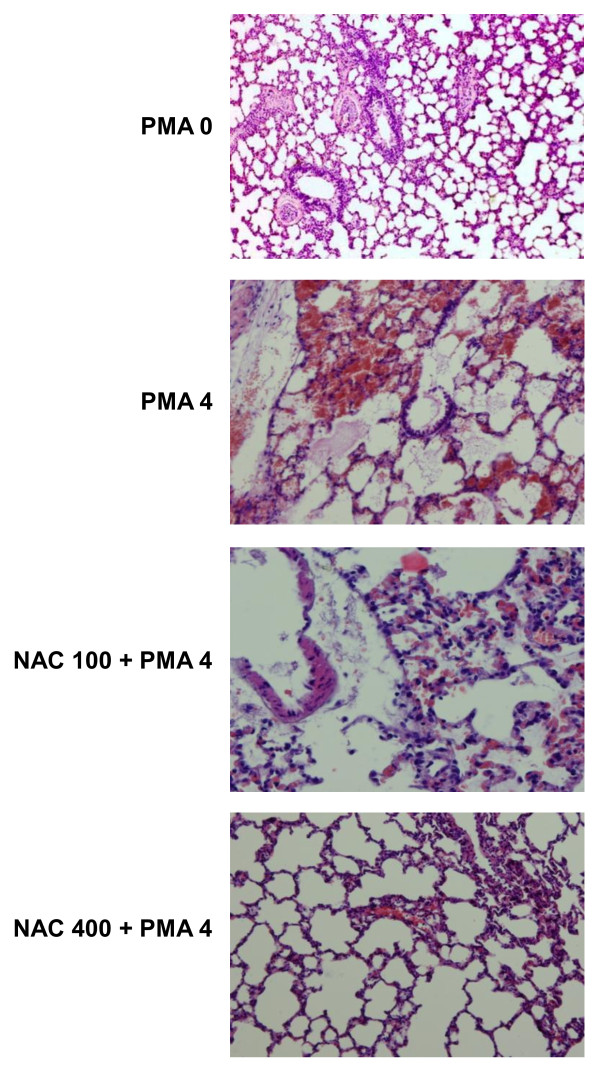
**Lung pathology in various groups**. PMA caused pulmonary edema and hemorrhage with inflammatory cell infiltration. NAC abrogated the pulmonary pathology in a dose-dependent fashion. To minimize redundance, only the effects of PMA 0 and 4 μg/g and NAC 100 and 400 mg/g on the PMA (4 μg/g) induced lung pathology are shown.

Figure 7 shows the histopathological micrographies of the lungs following PMA at 4 μg/g. The pulmonary pathological changes included edema and hemorrhage with infiltration of inflammatory cells. The pulmonary pathology was diminished or prevented by coadministration of NAC. The protective effects of NAC were operated in a dose-dependent fashion. PMA dose-dependently increased the LIS, NAC reduced the LIS caused by PMA. The reductions were also dose-dependent (Table 3).

**Table 3:** The lung injury score (LIS)

		LIS
PMA	0	0.12 ± 0.04
PMA	4	5.82 ± 0.22*
NAC	0	5.74 ± 0.24
NAC	100	2.38 ± 0.14^§+^
NAC	200	1.46 ± 0.12^§+^
NAC	400	0.24 ± 0.06^§+^

Values are means ± SEM (n = 10). The data collections, abbreviations and statistical symbols are the same as Table 1.

**Figure 8:**
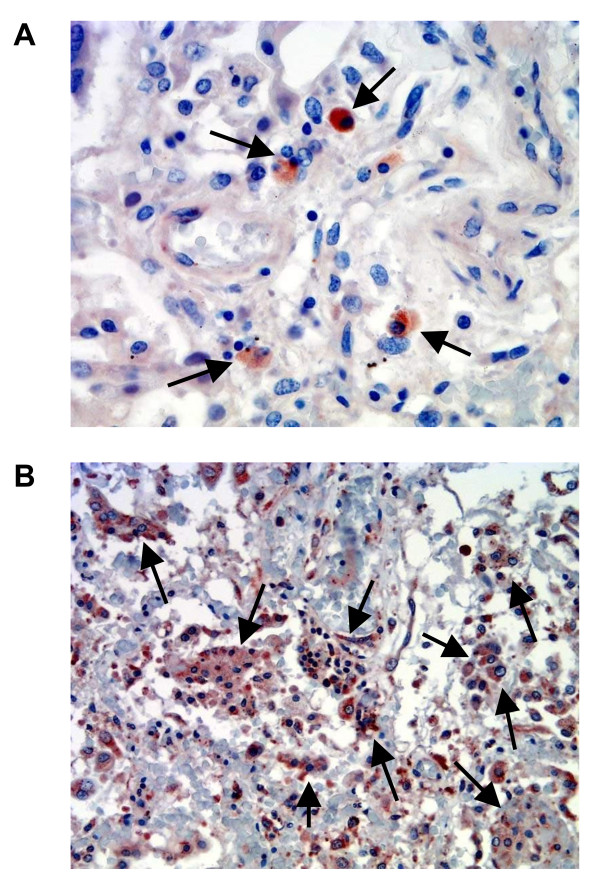
**Expression of inducible nitric oxide synthase (iNOS) in endothelial cells (A) and alveolar macrophages (B, arrows)**. Antigen retrieval immunohistochemical stain of lung tissue sections was used (original magnification ×200).

Immunohistochemical stain with antigen retrieval technique revealed the presence of iNOS-positive cells in alveolar macrophages and endothelial cells. The number of iNOS-producing cells seemed to be more abundant in macrophages than endothelial cells (Figure 8). It appeared that the number of iNOS-staining macrophages and endothelial cells was decreased by treatment with NAC (data not shown).

## Discussion

Previous studies have reported that niacinamide exerts protective effects on the acute lung injury induced by various challenges including endotoxin, ischemia/reperfusion, and oxidative stress [7,9,20,35-38]. In the present investigation, we found that this cytoprotective agent effectively abrogated the lung injury caused by phorbol myristate acetate in isolated lungs. Niacinamide diminished the pulmonary hypertension and increase in microvascular permeability following PMA introduction into isolated lungs. This B complex compound decreased ATP, while increased PARP. PMA also induced the upregulation of iNOS and eNOS. NAC mitigated the upregulation. Neutrophil activation by PMA led to releases of neutrophil elastase, myeloperoxidase and malondialdehyde in plasma and lung perfusate. NAC abrogated the biochemical changes caused by PMA. The PMA-induced ALI was associated with severe pulmonary edema and hemorrhage with inflammatory cell infiltration. Coadministration with NAC attenuated the lung pathology. NAC reduced the lung injury score after PMA. The results indicate that PMA exerts a detrimental role. The beneficial effects of NAC are dose-dependent.

Immunofluorescent staining revealed that PMA produced marked increases in nitrotyrosine and iNOS activies. The PMA effects on the nitrotyrosine and iNOS in lung tissues were mitigated by NAC in a dose-dependent fashion. NO is a free radical. The formation of nitrotyrosine and peroxynitrate by NO conjugation with reactive oxygen radicals such as peroxide and superoxide is extremely toxic to the lung in sepsis, ischemia-reperfusion, infections and phorbol myristate acetate as well [21,31,32,35-41].

In the lungs, PARP plays a key role in the microvascular platelet-endothelial cell interaction induced by endotoxin, acute lung inflammation following intratracheal administration of endotoxin, induction of asthma, an leukocyte recruitment in systemic endotoxemia [6,42,43]. These cellular interactions, tissue inflammatory changes and subsequent alterations in adhesion molecules are likely to be the fundamental basis for the pathogenesis of lung injury. The present study revealed that PMA increased the PARP activity, while reduced the ATP content. NAC attenuated the PMA-induced increase in PARP activity and restored the ATP content. In the regard, NAC exerts not only cytoprotective actions, but also anti-inflammatory effects with restoration of ATP.

Pharmacological inhibition and gene knockout mutants of PARS or PARP have become a new approach for the experimental therapy of various disorders, such as endotoxin shock, stroke, I/R injury, lung inflammation and others [42-44]. Activation of PARS or PARP produces cytotoxicity and subsequent cell death and organ dysfunction. The results of the present study demonstrated that treatment with NAC abrogated ALI induced by PMA in lungs.

In addition to the role of PARP in ALI due to various causes, iNOS may also be a crucial factor in lung damage. In endotoxin-induced lung damage, many studies have provided evidence to suggest that NO production through iNOS is harmful to the lungs in various species and different causes [41-46]. In patients with lung inflammation or injury, the iNOS expression and nitrotyrosine activity were increased [31,32]. The iNOS upregulation was found in subjects suffering from leptospirosis and scrub typhus [34,47]. PARP was involved in nuclear factor (NF)-κB expression and thereby activated NOS. ADP ribosylaion is required to activate NF-κB-mediated iNOS gene transcription [48,49]. Remick et al. [50] also suggested that ADP ribosylation was crucial in the signaling pathway which leads to NOS mRNA expression. PARP inhibitors prevent NOS induction, iNOS mRNA expression and TNF_α _release in interferon- and lipopolysaccharide-stimulated macrophages. After inhibition of PARP, iNOS expression, iNOS activity and NO production were reduced [6]. PARP inhibition also reduced the production of peroxynitrite, and prevented the presence of nitrotyrosine in the tissue [51]. It is likely that iNOS is involved in the pathogenesis of tissue injury following endotoxemia, ischemia-reperfusion and other challenges in various organs. In the present study, Real-time PCR and immunofluorescent staining revealed that PMA upregulated iNOS expression. The upregulation was attenuated by cotreatment with NAC.

Immunohistochemical stain observed the major sites of iNOS-producing cells were the alveolar macrophage and endothelial cells. The present study strongly suggests that NO production through the iNOS isoform plays a deleterious role in the PMA-induced lung injury. NAC possesses protective effects on the inflammatory responses to PMA. In this regard, the B-complex agent may act as a NOS inhibitor. Furthermore, ATP reduction and PARP increase caused by PMA could be the pathogenic mechanism of this neutrophil activator.

We are probably the first to demonstrate that the iNOS-positive cells following neutrophil activation by PMA reside mainly in the alveolar macrophages and endothelial cells. A recent study from our laboratory using electron microscopy has revealed the PMA caused endothelial damage [21]. The sites of cells that produce NO are similar to those observed in patients with lung infections [34,47]. We may propose that NO production through the iNOS-producing cells accounts for the lung injury and subsequent changes. In animals with sepsis, the NO production through the iNOS isoform was generated from the lung [20,21,52]. The present investigation further identified the sources of iNOS-positive cells.

Neutrophil activation and recruitment are an important step in the pathogenesis of ALI or acute respiratory syndrome (ARDS). We have provided evidence to show that PMA increases the neutrophil elastase, myeloperoxidase and malondialdehyde in lung perfusate. NAC significantly attenuated the increases in neutrophil derived mediators. The effects of NAC were operated in a dose-dependent fashion. Clinical investigations and trials are needed for the therapeutic application of this compound of B complex.

## Conclusion

In conclusion, neutrophil activation by PMA causes ALI and associated changes. NO release through the iNOS-positive cells is detrimental to the lung. The major sites of NO production are in the alveolar macrophages and endothelial cells. Inhibition of iNOS and/or PARP with NAC is likely the key mechanism of the protective effects of NAC on the PMA-induced lung injurious abnormalities.

This study investigated the effects and mechanisms of action of niacinamide (B complex) on the acute lung injury produced by a neutrophil activator, phorbol myristate acetate. The findings may have therapeutic values in clinical conditions of acute respiratory distress syndrome.

## Conflict of interests

The authors declare that they have no competing interests.

## Authors' contributions

CCL contributed in part to the study design. He conducted most of the animal experiments. NKH was in charge of the data collection and analysis. HLL performed the statistical analysis with NKH. She also contributed to the study protocol. HIC was the principal investigator. He obtained the grant and coordinated the study, and responsible for the completion and revision of the manuscript. All authors have read the final manuscript, and approved the submission to Journal of Biomedical Science.
